# CX3CL1 (Fractalkine) Protein Expression in Normal and Degenerating Mouse Retina: *In Vivo* Studies

**DOI:** 10.1371/journal.pone.0106562

**Published:** 2014-09-05

**Authors:** Marina Zieger, Peter K. Ahnelt, Pavel Uhrin

**Affiliations:** 1 Department of Neurophysiology and Neuropharmacology, Center of Physiology and Pharmacology, Medical University of Vienna, Vienna, Austria; 2 Department of Vascular Biology and Thrombosis Research, Center of Physiology and Pharmacology, Medical University of Vienna, Vienna, Austria; 3 Department of Ophthalmology and Gene Therapy Centre, University of Massachusetts Medical School, Worcester, Massachusetts, United States of America; Universidade Federal do ABC, Brazil

## Abstract

We aimed to investigate fractalkine (CX3CL1) protein expression in wild type (wt) retina and its alterations during retinal degeneration in mouse model (rd10) of *retinitis pigmentosa*. Forms of retinal protein CX3CL1, total protein and mRNA levels of CX3CL1 were analyzed at postnatal days (P) 5, 10, 14, 22, 30, 45, and 60 by Western blotting and real-time PCR. Cellular sources of CX3CL1 were investigated by *in situ* hybridization histochemistry (ISH) and using transgenic (CX3CL1cherry) mice. The immunoblots revealed that in both, wt and rd10 retinas, a membrane integrated ∼100 kDa CX3CL1 form and a cleaved ∼85 kDa CX3CL1 form were present at P5. At P10, accumulation of another presumably intra-neuronal ∼95 kDa form and a decrease in the ∼85-kDa form were observed. From P14, a ∼95 kDa form became principal in wt retina, while in rd10 retinas a soluble ∼85 kDa form increased at P45 and P60. In comparison, retinas of rd10 mice had significantly lower levels of total CX3CL1 protein (from P10 onwards) and lower CX3CL1 mRNA levels (from P14), even before the onset of primary rod degeneration. ISH and mCherry reporter fluorescence showed neurons in the inner retina layers as principal sites of CX3CL1 synthesis both in wt and rd10 retinas. In conclusion, our results demonstrate that CX3CL1 has a distinctive course of expression and functional regulation in rd10 retina starting at P10. The biological activity of CX3CL1 is regulated by conversion of a membrane integrated to a soluble form during neurogenesis and in response to pathologic changes in the adult retinal milieu. Viable mature neurons in the inner retina likely exhibit a dynamic intracellular storage depot of CX3CL1.

## Introduction

*Retinitis pigmentosa* (RP) constitutes a large, heterogeneous group of inherited retinal neurodegenerative conditions. The phenotype is characterized by an initial rod-cone dystrophy followed by an aberrant remodeling of the surviving retina that corrupts visual processing [1], [2]. Retinal remodeling is a universal pathologic process subsequent to retinal degenerative disease that results in deafferentiation of the neural retina from photoreceptor input. Downstream neuronal elements respond to loss of input with a phased revision of retinal structure and function found at the molecular, synaptic, cell, and tissue levels and involving all cell classes in the retina, including neurons and glia [3], [4].

Within the nervous tissue, neurons, express “on and off“ signals to maintain their milieu through the release of factors that control innate immune cell function, including microglia [5]–[8]. Microglia are resident cells of the nervous tissue involved in regulatory processes critical for development, maintenance of the neural environment, injury and repair and, as such, are sensors of events occurring within their immediate environment [9]. Degenerating neurons release several signaling molecules, including nucleotides, cytokines and chemokines, to recruit microglia and enhance their activities [10], [11]. It was demonstrated that all retinal microglial cells express a receptor for CX3CL1, a chemokine receptor 1 (CX3CR1) [12].

Fractalkine (also known as neurotactin, fractalkine ligand 1, CX3CL1) is expressed as multi-domain membrane-spanning molecules consisting of a chemokine domain with CX3C motif, a flexible highly glycosylated mucin-like stalk, a transmembrane domain and a short cytoplasmic domain [13]. It is one of the neuroimmune regulatory proteins that is abundantly expressed on neurons and signals through its single G-protein-coupled receptor (CX3CR1), exclusively on microglia [14]–[20]. Endogenous expression of CX3CL1 produces a membrane integrated monomeric isoform [21] that can be cleaved by several proteases including a disintegrin and metalloproteinase (ADAM) family, ADAM-10, ADAM-17 [22]–[24] and cathepsin S [25]. Such cleavage yields a soluble isoform of CX3CL1, comprising of the mucin-like stalk and the chemokine domain. Both isoforms of CX3CL1 have been shown to ligate CX3CR1 [22], [23], [26], [27]. CX3CL1 directly induces various microglial functions including migration [14], proliferation [28], inhibition of Fas-ligand-induced cell death [29], glutamate-induced neurotoxicity [30], [31] and inhibition of proinflammatory cytokines production [5], [16]. Recently, it was shown that the soluble form of CX3CL1 directly enhances microglial clearance of degenerated neuronal debris [31]. Thus, CX3CL1 represents a unique chemokine that contain properties of both chemoattractant and adhesion molecules, since it can exist either as a soluble protein or a membrane integrated molecule, playing a pivotal role in signaling between degenerating neurons and microglia [32]. However, the individual signaling functions of each CX3CL1 isoform under physiological and neurodegenerative conditions are not fully understood.

Strong evidence indicates that soluble fractalkine prevents N-Methyl-D-aspartate-neurotoxicity [18], [27], [33]. It controls glutamatergic activity by inhibiting excitatory postsynaptic currents [33], [34], and negatively regulates neuronal migration; while promoting the adhesion to extracellular matrix [35]. However, the extent, to which each of these mechanisms is involved in the physiological role of fractalkine, may vary between normal, inflammatory and neurodegenerative conditions, at different stages of life and/or during diseases states [36]–[38].

In contrast to the growing amount of research data from the brain, not much is known about the potential roles of CX3CL1 in the retina. The purpose of the present study was therefore to analyze in detail, the CX3CL1 expression in normal and degenerating mouse retina. As a model of autosomal recessive RP, we used in our study rd10 mice bearing a spontaneous missense point mutation in exon 13 of the beta-subunit of the rod cGMP phosphodiesterase gene, a member of the phototransduction cascade [39]. Mutations in the cGMP phosphodiesterase gene have been identified in approximately 5% of cases in human patients with autosomal recessive RP [40].

In order to elucidate possible alterations of the cellular communication between neurons and microglia involving cytokines and chemokines, such as fractalkine, we characterized forms of CX3CL1 protein, estimated total protein and mRNA levels of CX3CL1 and the cellular source of CX3CL1 in the retinas at normal and pathological states.

We focused on a time window from birth to young adulthood, encompassing in rd10 mice onset and late stages of rod and cone cell degeneration along with the early phase of second and third order neuron remodeling [39], [41]–[45].

## Materials and Methods

### Animals

Rd10 homozygous (rd10) mice in C57BL/6J background were obtained from Charles River Laboratories (Germany). Wild-type (wt) background control C57BL/6J mice were imported from Jackson Laboratories (USA). Rd10 and C57BL/6J mice were intercrossed to obtain heterozygous animals from which homozygous rd10 mice and control wt littermates were obtained. These animals were used as breeders for generating the mice used in the experiments. Additionally, eye samples of CX3CL1cherry∶CX3CR1gfp transgenic mice expressing red and green fluorescent reporter genes under the respective control of the CX3CL1 and CX3CR1 promoters [46], [47] were also characterized which were kindly provided by Steffen Jung, The Weizmann Institute of Science, Rehovot, Israel.

Rd10 and wt mice were kept under 12-hour light/dark cycle with lights on at 7:00 am in a temperature (22±1°C) and humidity (55±5%) controlled room. All cages were placed on the lower shelf of an IVC rack (Maximizer, Ehret, Austria) with reduced light illuminance of 30 Lux, as measured with Gossen MAVOLUX 5032 B USB Digitales Luxmeter (Elektrohandel Thiele, Germany). The cages contained wood shavings as bedding and a plastic tube for environmental enrichment. Mice were fed ad libitum with standard chow and supplied with mildly acidified water. For collection of retinas and brain samples, mice at postnatal day 5 (P5) were sacrificed by decapitation. Older ones (at P10, P14, P22, P30, P45 and P60) were pre-treated by CO_2_ asphyxiation, immediately followed by cervical dislocation. The animals' health status was monitored throughout the experiments by a health surveillance program according to Federation of European Laboratory Animal Science Associations (FELASA) guidelines.

100 mice were utilized for this study and 86 were included and completed. 14 animals were excluded because of instrumentation or technical failure during sample preparation.

### Ethics Statement

All procedures of animal handling conformed to the European Union guidelines for the use of laboratory animals (Directive 2010/63/EU of the European Parliament and of the Council) and to the ARVO Statement for the Use of Animals in Ophthalmic and Vision Research. Animal care and all experimental procedures were approved by the Animal Experimental Committee of the Medical University of Vienna, and by the Austrian Ministry of Science (License No. BMWF-66.009/0222-II/3b/2013). Although Rd10 mice exhibited postnatal retinal degeneration, due to their low-grade phenotype no special requirements on amelioration of suffering of these mice were a priori required.

### Tissue Preparation

The animals' eyes were rapidly enucleated, hemisected and, after dissection of sclera-choroid tissue with fine forceps, the retinas were detached from the pigment epithelium. For real-time PCR the right and left isolated retinas of each animal were pulled together, placed into screw cap cryotube and were immediately frozen at −80°C. For Western blot studies, the retinas were initially prepared in the same way as for real-time PCR. Both left and right retinas of each animal were collected separately to compose the duplicates of a single mouse. Also the brains were rapidly removed, immersed into an ice-cold sterilized 0.01 M PBS, and the hippocampal dentate gyrus subregion was dissected out. Retina and brain tissues were placed with fine clean forceps into Eppendorf microcentrifuge tubes, then snap frozen in liquid nitrogen and stored at −80°C. For histology and *in situ* hybridization the eyes were rapidly enucleated, fixed in 4% paraformaldehyde for 1 hour, then conjunctiva, cornea, crystalline lens and vitreous were dissected and discarded. After fixation, the tissues were washed with PBS. Eye-cups and rostro-caudal coronal 5 mm thick blocks of mouse brains containing the hippocampal area were either frozen for cryosectioning or embedded in paraffin.

### Western Blot Analysis

The retinas and brains were homogenized by BioVortexer homogenizer in ice-cold lysis buffer (10 mM Tris-HCl, pH 7.5; 67 mM NaCl) containing 1% SDS, 0.1 mM EDTA, protease inhibitor cocktail (Roche Diagnostic, Basel, Switzerland, Cat.# 04693124001), and phosphatase inhibitors 1 mM sodium orthovanadate, 1 mM sodium fluoride, 0.5 mM tetrasodium diphosphate. After 30 minutes incubation on ice, lysates were cleared by centrifugation, and protein concentration was determined by BSA protein assay (Pierce Biotechnology, Rockford, IL, USA). Equal amounts of protein (10 µg/sample/lane) were loaded onto Tris-Glycine 12% SDS PAGE run under reducing conditions. As a marker, Spectra Multicolor Broad Range Protein Ladder (Cat.# 26634, Thermo Fisher Scientific, Waltham, MA, USA) was used. Upon electrophoretical transfer to PVDF membranes (Cat.# T830.1, Carl Roth GmbH, Germany), blots were incubated for 1 hour with 5% non fat dry milk in Tris-buffered saline containing 0.1% Tween 20 and incubated overnight at 4°C with a polyclonal rabbit anti-CX3CL1 antibody raised against the e. coli-expressed extracellular domain (aa1-85) of human fractalkine and cross reacting with mouse and rat CX3CL1 (0.3 µg/mL, Cat.# ab25088 Abcam, Cambridge, UK). Upon treatment with HRP conjugated donkey anti-rabbit IgG ECL antibody (1∶4000, GE Healthcare, Chalfont St Giles, Buckinghamshire), enhanced chemiluminescence (ECL) analysis (GE Healthcare Europe GmbH) was imaged with FluorChem HD2 detection system (Alpha Innotech, San Leandro, CA, USA). For quantification, stripped blots were re-probed with mouse monoclonal anti-glyceraldehyde-3-phosphate dehydrogenase (anti-GAPDH) primary antibody (0.2 µg/mL, Cat.# AM4300, Life Technologies, Carlsbad, CA, USA) followed by secondary HRP conjugated anti-mouse IgG antibody (1∶10000, GE Healthcare Europe GmbH). All experiments were performed in biological (five mouse retinas) and technical (five replicates per mouse) quintuplicates. Densitometry was performed using Image J analysis software version 1.44, developed at the U.S. National Institutes of Health and available on the internet at http://rsb.info.nih.gov/nih-image/.

### Antibody Control Experiments

The polyclonal rabbit anti-CX3CL1 antibody was tested for signal specificity with antigen-peptide neutralization (competition) assay. The preadsorption was performed on identical immunoblots. Antibody was diluted in TBST. The compositions of the blocking solution and the final antibody concentrations were as stated above. Each of these antibody solutions was then divided in 2 aliquots to obtain pairs of identical solutions. A positive control for the antibody detection, consisting of an ∼70-kDa form recombinant human fractalkine peptide (aa 25-100), lacking the signal peptide, mucin-like stalk, both transmembrane and the carboxy-terminal domains (PeproTech Cat.# 300-31), was added at 0.6 µg/mL to one aliquot from each pair. The immunoblots were then incubated overnight at 4°C before being processed for immunolabeling as described above.

### Real-time PCR

Total RNA was isolated by standard procedure using Trifast (PeqLab Biotechnology, Erlangen, Germany) and Precellys 24 homogenizer (PeqLab Biotechnology), followed by reverse transcription using the MuLV-reverse transcriptase I in the presence of RNAse inhibitor and oligo dT16 primers (Life Technologies). Real-time PCR was accomplished in Step One Plus Real-Time PCR Cycler (Life Technologies) using FastStart SYBR Green Master Mix (Life Technologies) and CX3CL1 specific primers (forward 5'-CGC GTT CTT CCA TTT GTG TA-3' and reverse 5'-CTG TGT CGT CTC CAG GAC AA-3' CX3CL1 primers resulting in a 169-bp amplicon) by normalizing to the expression of β2-microglobulin (5'-GAT GAG TAT GCC TGC CGT GTG-3' and 5'-CAA TCC AAA TGC GGC ATC T-3' resulting in a 114-bp amplicon).

### Immunohistochemistry

At least three rd10 and wt mice were studied at selected time points. Eye samples derived from transgenic (CX3CL1cherry: CX3CR1gfp) mice were collected in a similar way. As controls, brain samples containing hippocampal dentate gyrus subregion were collected and prefixed as above. After fixation, tissue samples were washed with PBS, submerged into 20% sucrose and mounted in Tissue-Tek O.C.T. Serial cryo-sections (10 µm) were permeabilized in PBS containing 0.5% Triton X-100, blocked in 10% BSA in PBS containing 0.25% Triton X-100 for 1 hour at room temperature and exposed to primary antibodies overnight at 4°C. Mouse monoclonal anti-mCherry antibody (1∶300, 1C51, Cat.# ab125096 Abcam, Cambridge, UK) and rabbit polyclonal anti-GFP antibody (1∶1000, Cat.#6556 Abcam, Cambridge, UK) were used to immunolabel mCherry and GFP expressing cells, respectively. After washing with PBS, sections were exposed to secondary antibodies for 1 hour at room temperature, then washed with PBS, counterstained with DAPI (Sigma-Aldrich, Saint Louis, MO, USA, Cat.# A3648) and mounted in Fluoro-Gel aqueous medium (Science Services GmbH, München, Germany). Microscopic analysis was performed either by Eclipse E600 (Nikon, Tokyo, Japan) or Axiovert 200 M (Carl Zeiss, Jena, Germany) microscopes. In the latter, image acquisition was enhanced using a Zeiss Apotome Confocal System. Immunohistochemical controls were performed by omission of either the primary or secondary antibodies.

### RNA probes (riboprobes)

The cellular localization of fractalkine mRNA was detected with digoxigenin-labeled single-stranded cRNA probes using *in situ* hybridization. ORF clone of *Mus musculus* CX3CL1 cDNA was subcloned into a plasmid pGEM-T vector (Sino Biological Inc., China, Cat.# MG50917-G) and propagated in *E. coli* strain (XL1-Blue supercompetent cells, Stratagene, USA, Cat.# 200236). The DNA was purified using the PureLink HiPure Plasmid DNA Midiprep Kit (Invitrogen, USA, Cat.# K2100-15), linearized with the appropriate restriction enzyme digestion and extracted from preparative agarose gel using PCR Clean-Up System (Promega, USA, Cat.# A9282). The antisense and sense strand negative control riboprobes were generated by *in vitro* transcription from 1 µg of the linearized DNA. Purified DNA templates were used for a transcription with SP6 (Cat.# P1085) and T7 (Cat.# P2077) RNA polymerases (Promega, USA) and DIG-RNA-labeling kit (Roche Diagnostics GmbH Applied Science, Vienna, Austria, Cat.# 11175025910), giving about 10 µg labeled RNA per µg of template. The riboprobes were purified through a Sephacryl S-300 MicroSpin column (GE Healthcare, Product code 27-5130-01) to remove unincorporated digoxigenin-labeled nucleotides. The incorporation of DIG label into the RNA probes was estimated on dot blot by using colorimetric assay, similar to the *in situ* hybridization detection method. Dots (1 µL) of the probe at serial dilutions were spotted on a nylon membrane, dried, and UV-linked for 2 min. Sense CX3CL1 cRNA was used as background control. To test for nonspecific binding of the secondary detection systems, hybridization was also performed in the absence of specific probes.

### Pretreatment of Sections for *In Situ* Hybridization

For paraffin sections slides were pre-coated with 2%, 3-aminopropyltriethoxysilane (Sigma-Aldrich, St. Louis, MO, USA, Cat.# A3648) and air-dried. After dewaxing, 3 µm sections were postfixed in 4% paraformaldehyde in 0.1 M phosphate buffer and treated with 0.2 M HCl. Proteinase K (10 µg/mL) (Sigma-Aldrich, St. Louis, MO, USA, Cat.# P2308) digestion was performed at 37°C in 0.5 M Tris-HCl buffer (pH 7.4) for 5 min. The enzymatic reaction was stopped with Tris-HCl buffer at 4°C. Nonspecific staining was prevented by acetylation (0.5% acetic acid anhydride in 0.1 M Tris-HCl buffer, pH 8.0) for 10 min. The sections were then dehydrated in graded ethanol and were rinsed in chloroform. Frozen 8 µm sections were air-dried and processed in similar way as paraffin sections.

### *In Situ* Hybridization

*In situ* hybridization on cryosections of retinal or brain samples or paraffin-embedded brain sections was performed as described previously [48], [49]. Briefly the probes were denatured, and the probe solution was pipetted onto the slides, then covered with a coverglass and placed on a hot plate at 95°C for 4 min. The hybridization solution contained: 47% deionized formamide, 2× SSC, 10% dextran sulfate, 0.01% sheared salmon sperm DNA (Sigma-Aldrich, St. Louis, MO, USA, Cat.# D7656), 0.02% SDS, and the labeled probes used in 1∶100 working concentration, as determined in dot-blots. Hybridization was performed at 65°C for 16–20 h. After hybridization, the sections were washed in 50% deionized formamide (N, N-dimethylformamide, Sigma-Aldrich, Saint Louis, MO, USA, Cat.# D4551) in 1× SSC at 55°C for 1 hour. Unsaturated binding sites were blocked with blocking reagent. Digoxigenin labeling was detected with alkaline phosphatase-conjugated anti-digoxigenin antibody at 1∶500 (antidigoxigenin AP, Fab fragments, Roche Diagnostics GmbH Applied Science, Cat.# 11093274910), in 0.5% blocking reagent (Roche Diagnostics GmbH Applied Science, Cat.# 11096176001), with 10% fetal calf serum. Development of the *in situ* hybridization was performed in 4-nitro blue tetrazolium chloride (Roche Diagnostics GmbH Applied Science, Cat.# 11585029001) and 5-bromo-4-chloro-3-indolyl phosphate (Roche Diagnostics GmbH Applied Science, Cat.# 10760994001) for 20 hours. The sections were counterstained by hemalaun and mounted into GelTol aqueous medium.

### Statistical Analysis

Values are presented as mean ± SEM with significance determined at p<0.05. Statistical significance was evaluated by Student's unpaired two-tailed *t* test, and by Pearson product-moment correlation coefficient using Graph Pad Software available on the internet at http://www.graphpad.com.

## Results

### CX3CL1 Protein Forms Revealed by Western Blotting

**Figure 1:**
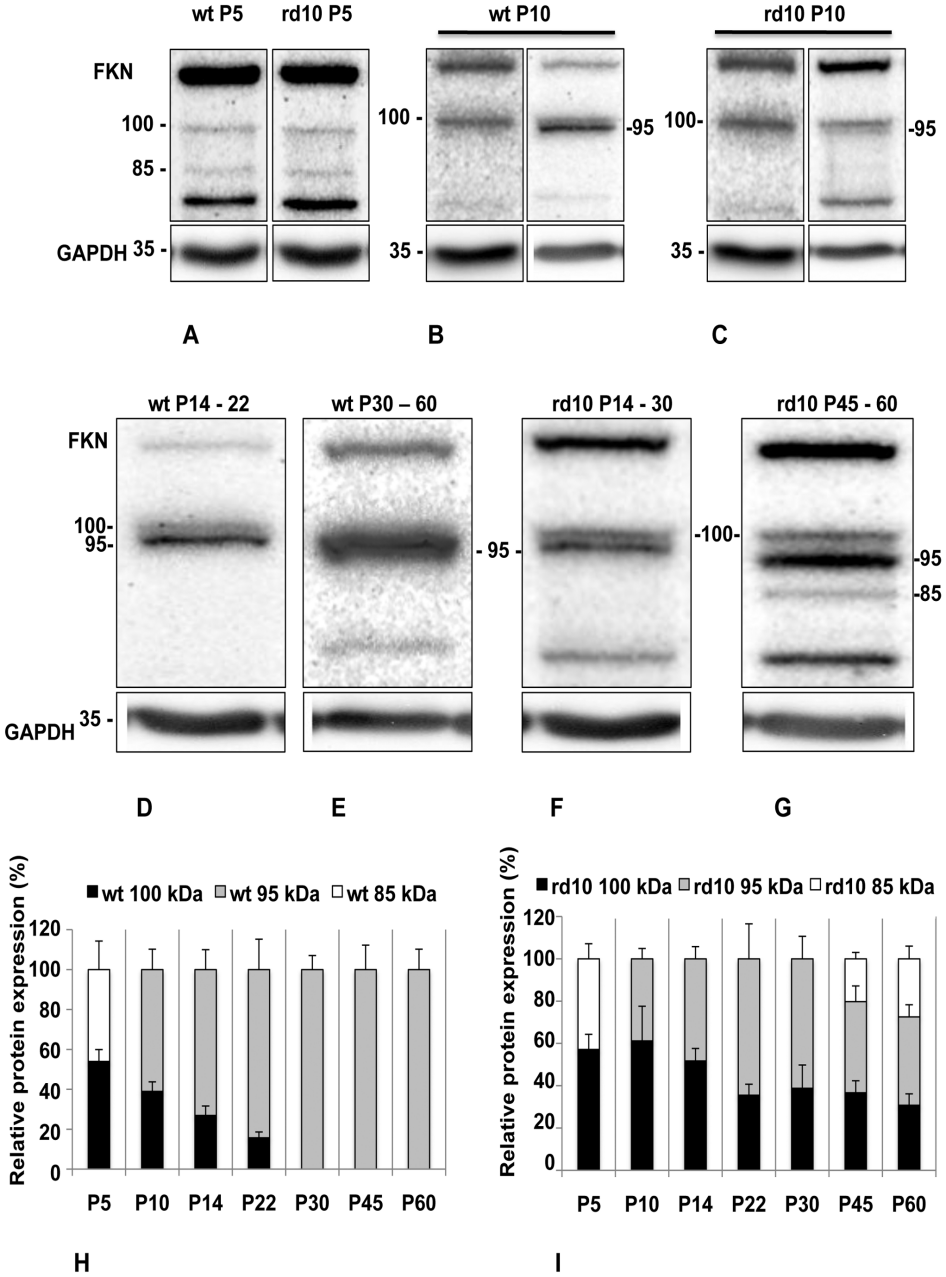
Western blot analysis of fractalkine protein expression in mouse neural retina at P5 until P60 being representative of five independent experiments. Blots were re-probed with anti-GAPDH antibody. (A). Fractalkine membrane integrated (100-kDa) and soluble (85-kDa) protein forms are present in both control (wt) and rd10 retina lysates during development at P5. (B). Accumulation of presumably intracellular (95-kDa) protein form is seen in both wt (B) and rd10 (C) developing neural retina at P10. Remarkably, no cleaved (85-kDa) protein form was associated with membrane integrated (100-kDa) form in both rd10 and wt retina lysates at P10. Both wt and rd10 retinas show lower to beneath detection level of 100-kDa form and higher level of 95-kDa form at P14 through P60 (D-G). Increased level of 85-kDa form is clearly detectable in rd10 retinas at P45 and P60 (G). Histogram showing relative percent levels of each of the three CX3CL1 protein forms in wt retina (H) and degenerating rd10 retina (I). Data are expressed as percent of densitometric arbitrary units. Values are mean ± SEM, (n = 5). In rd10 retinas, positive correlation between the relative levels of a ∼100-kDa and corresponding 85-kDa bands was found at P45 and P60 (Pearson product-moment correlation coefficient r = 0.683, n = 12, p = 0.014 in RD10 retina at P45 and r = 0.882, n = 9, p = 0.0017 at P60) as well as for the relative levels of a 100-kDa and 95-kDa bands (r = 0.928, n = 11, p<0.0001 at P14, r = 0.861, n = 9, p = 0.0029 at P22, r = 0.892, n = 9, p = 0.0012 at P30, r = 0.966, n = 12, p<0.0001 at P45, and r = 0.628, n = 12, p = 0.0288 at P60 in rd10 retina). The degree of association between the levels of 95 kDa and 85 kDa protein band was r = 0.712, n = 12, p = 0.0094 at P45 and r = 0.907, n = 9, p = 0.0007 at P60. It was not, however, possible, to make similar analysis for the wild type retina samples, as the level of the ∼95-kDa protein form was the highest and the levels of cleaved and full-length forms were far below the limit of detection.

Using an antibody recognizing the extracellular domain of CX3CL1, three CX3CL1 bands of molecular weights ∼100 kDa, ∼95 kDa and ∼85 kDa were detected. At developmental day 5, fractalkine full-length ∼100 kDa and cleaved ∼85 kDa protein forms were present in both wt and rd10 retinal lysates at comparable levels (Fig. 1A). As development of the retina progressed (at P10), the tendency of retinal neurons toward accumulation of another ∼95 kDa protein form in both wt (Fig. 1B) and rd10 (Fig. 1C) became evident. Simultaneously, at P10 the ∼85 kDa protein form had dropped to the levels below detection limit in both rd10 and wt retinal lysates. From P14 through adulthood, a band at ∼95 kDa was dominant in wt (Fig. 1D and E) and was also highly expressed in rd10 retinas (Fig. 1F and G). At P45 and P60 the shorter ∼85-kDa form was clearly detected again in rd10 retinas indicating increased proteolytic cleavage compared to the wt retinas (Fig. 1G).

### Relative occurrence of CX3CL1 Protein Forms

Analysis of the expression of CX3CL1 protein forms, as percentage of the total protein level (Fig. 1H-I), revealed that full-length ∼100 kDa and cleaved ∼85 kDa forms are present at comparable levels in wt and rd10 retinas at P5 (p<0.05). From P10 through P60, the initial balance is changing toward an increase of a ∼95 kDa form in normal (wt) retinas (p<0.05). Remarkably, the ∼95 kDa appeared to be the only form clearly detected in wt retina at P30 to 60. Interestingly, from P10 through P60, the ∼95 kDa protein form in rd10 retina is present at lower percentage rate than in wt (p<0.05). When the ∼85 kDa band increased in rd10 at P45–60, it appeared that ∼95 kDa and ∼100 kDa forms are present at comparable levels (p<0.05).

To assess the relationship between the three forms of fractalkine protein, we computed a Pearson product-moment correlation coefficient (r). We showed a positive correlation among the detected CX3CL1 bands in rd10 retinas (as summarized in Fig. legend 1). We further proved the specificity of anti-CX3CL1 antibody used in Western blots and revealed the absence of sex-related differences in retinal samples of rd10 and wt mice (Fig. S1). Finally, we demonstrated the suitability of the usage of GAPDH as endogenous control for CX3CL1 protein expression normalization in Western blots (Fig. S2). Even though, using a set of well-characterized antibodies and lectins, we observed, consistently with other studies [39], [41], [43]–[45], [50], [51] the expected degeneration over time of photoreceptors and second order neurons in the retina of rd10 mice (data not shown), the relative density of GAPDH remained roughly constant in total retinal protein homogenates. Thus, we can conclude that usage of GAPDH as internal control assured a reliable detection of alterations in levels of CX3CL1 protein in Western blots of retinas of rd10 and wt mice.

### Decrease of both, CX3CL1 Protein and mRNA in Rd10 compared to Wt Retinas

**Figure 2:**
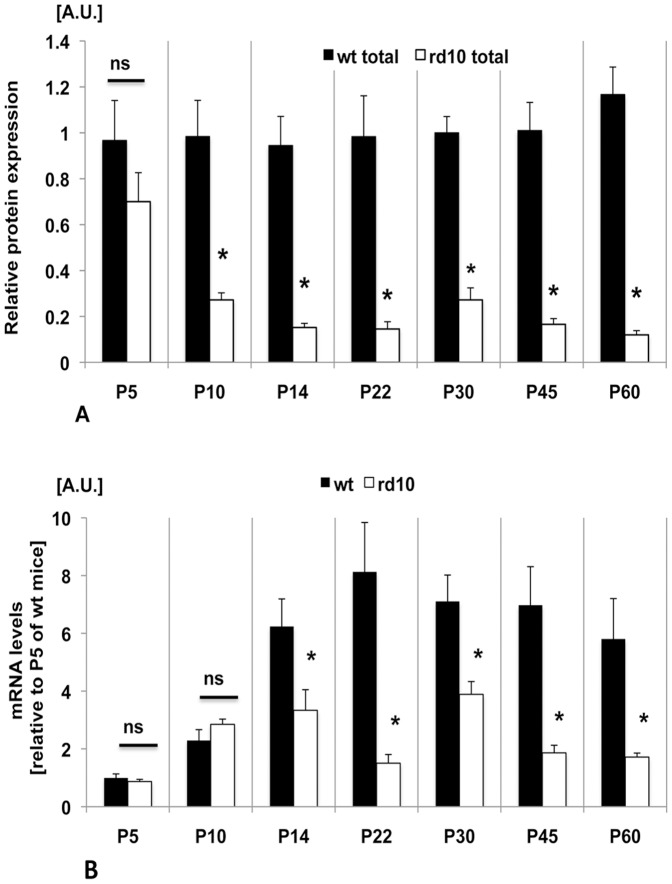
Comparison of total CX3CL1 protein and mRNA expression levels in rd10 and wt retinas. (A). Western blot analyses of total CX3CL1 protein levels in rd10 retinas compared to wt retinas determined in five independent experiments. Total protein levels in rd10 retinas are significantly decreased at P10 and remain low at all time points examined. Results (mean ± SEM, n = 5) are expressed as fold change over control in densitometric arbitrary units. *P<0.0001 versus control. (B). Expression of CX3CL1 mRNA in wt and rd10 retinas at P5, P10, P14, P22, P30, P45 and P60, determined by real-time PCR normalized to β-microglobulin levels. Comparable CX3CL1 mRNA expression level in control wt and rd10 retinas at P5, P10 and lower levels at P14 and later time-points in rd10 retinas compare with wt retina. Values are mean ± SEM (n = 5–7; *p<0.05; ns: not significant).

CX3CL1 total protein in wt retinas was relatively low at P5, increased at P10, peaked at P14 and remained at relatively high constant levels at P22, P30, P45 and P60. In rd10 retinas, levels of CX3CL1 protein were comparable to wt retinas at P5. However, they decreased at P10 and remained considerably low in comparison to wt retinas at all subsequent analyzed time points (P14, P22, P30, P45 and P60) (Fig. 2A). Thus we can conclude that levels of CX3CL1 in rd10 mice were significantly lower, in comparison with wt retinas, as early as postnatal age, at P10.

CX3CL1 mRNA levels revealed that in wt retinas mRNA showed an increase at P10 as compared to P5. It further increased at P14 and remained relatively constant until P60. In rd10 retinas, CX3CL1 mRNA levels were comparable to wt retinas only at P5 and P10 and from P14 onwards, they were significantly lower compared with wt retinas (Fig. 2B). Overall these results demonstrate that CX3CL1 mRNA (from P14), and total protein levels (from P10), are significantly lower in rd10 compared to wt retinas.

### Localization of CX3CL1 mRNA Expressing Cells by *in situ* hybridization

**Figure 3:**
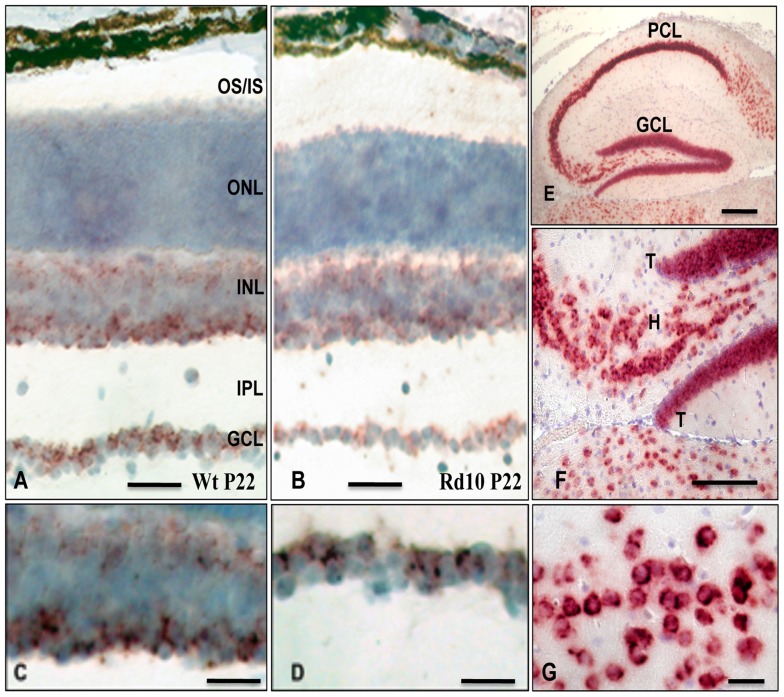
Localization of CX3CL1 mRNA expressing cells in the retina and brain of wt and rd10 mice. (A-D). Light micrographs of radial cryosections through mouse central retina probed with a DIG-labeled anti-sense CX3CL1 riboprobe. In both, wt (A) and rd10 (B) mouse retinas, CX3CL1 mRNA signal is present exclusively in the neurons of the ganglion cell layer (GCL) and the inner- and outermost areas of the inner nuclear layer (INL). The outer nuclear (photoreceptor) layer is devoid of CX3CL1 signal at the time points examined (P22 and P30). Enlargements showing juxtanuclear localization of mRNA in the cells of inner nuclear (C) and ganglion (D) cell layers in wt mouse retina. (E-G). Distribution of CX3CL1 mRNA expressing neurons in wt mouse hippocampus. Light micrographs of a paraffin section used as positive control. (E). Numerous CX3CL1 mRNA-positive neurons are located in all hippocampal subfields of the pyramidal (PCL) and in the dentate granule cell layers (GCL). (F). Higher magnification of the dentate gyrus tips and of hilus. (G). Enlargement showing high levels of CX3CL1 mRNA expression within the grey matter of the cerebral cortex. PCL, pyramidal cell layer; GCL, layer of ganglion cells in dentate granule cell layer; H, hilus; T, dentate gyrus tips. Cells expressing CX3CL1 mRNA are visualized as of brown product accumulations in cryosections and red-brown in paraffin sections. The different color of nuclei staining (blue in cryosections and purple in paraffin sections) is due to a hemalaun counterstaining. Scale bars represent: 50 µm (A, B), 35 µm (C, D), 250 µm (E), 125 µm (F) and 25 µm (G).

To define the cellular source of CX3CL1 in the retina, we accomplished *in situ* hybridization experiments on retinal tissue sections of rd10 and wt mice. The strongest labeling was displayed in a subpopulation of neurons within the ganglion cell layer, while the outer nuclear (photoreceptor) layer was devoid of CX3CL1 expression in both wt and rd10 retinas at all examined time-points. The analyses revealed CX3CL1 mRNA expression in neurons of inner- and outermost-subtiers of the inner nuclear layer and in the ganglion cell layer in both wt and rd10 retinas (Fig. 3A-D), while no expression was detected in the distal retina or in cells associated with the retinal vasculature. In control brain samples of wt mice, high levels of CX3CL1 mRNA were detected in pyramidal cells within the hippocampal formation (Fig. 3E-F) and the grey matter of the cerebral cortex (Fig. 3G). As expected, parallel paraffin brain and retinal cryosections hybridized with a control sense-transcribed CX3CL1 cRNA probe of equal specific activity did not reveal signals above background levels (data not shown). Overall, our *in situ* hybridization experiments revealed an unambiguous neuronal expression of CX3CL1 mRNA in rd10 mouse retina at P22 and P30.

### Analysis of CX3CL1 Expression in Control Transgenic Mice

**Figure 4:**
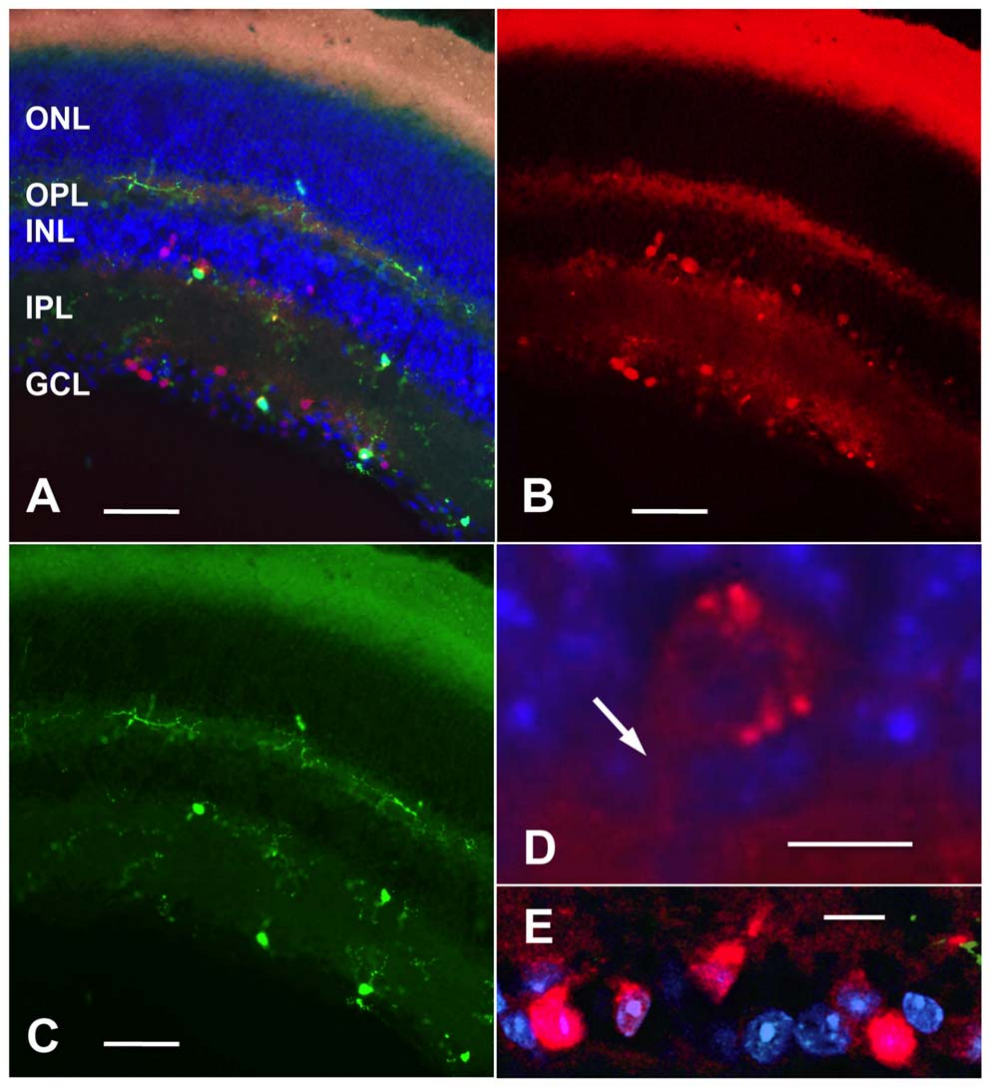
Regionally restricted CX3CL1 expression in the retina of adult CX3CL1 cherry: CX3CR1gfp mouse (A-E). Fluorescent light microscopy analysis of a vertical retina cryosection (A) showing both CX3CL1/Cherry (detected in rhodamine channel, red) (B) and CX3CR1/GFP (detected in FITC channel, green) (C) reporters expression. CX3CR1/GFP signal is assigned to microglial cells with cell bodies and dendritic ramifications in three sublayers (outer plexiform layer, OPL, inner plexiform layer, IPL and ganglion cells layer, GCL). CX3CL1/Cherry fluorescent cells are found exclusively in ganglion cell and inner nuclear layer. No fluorescent cells were observed in outer nuclear layer (ONL). Enlargements show strong Cherry signal in a presumable amacrine cell body in inner nuclear (D, arrow points to dendritic base) and ganglion cell (E) layers, indicating high level of the CX3CL1 chemokine promotor activity in neurons. Cryosections are counterstained with DAPI nucleic acid stain (blue). Distribution of the Cherry reporter positive cells in the retina of CX3CL1cherry: CX3CR1gfp mice coincides mostly with results obtained in *in situ* hybridization studies. Of note, membrane/lipid inner and outer photoreceptor segment layer exhibit nonspecific fluorescence. Scale bars represent 50 µm (A, B and C), and 10 µm (D, E).

To further characterize CX3CL1 and CX3CR1 expression pattern in the retinas, we made use of transgenic (CX3CL1cherry∶CX3CR1gfp) mice [46]. Fluorescence analysis of cryosections of retinas derived from these revealed a regionally restricted CX3CL1/Cherry signal. It was present exclusively in the ganglion cell and inner nuclear layers of the retinas, indicating high level of the CX3CL1 promoter activity in at least a portion of these neurons (Fig. 4, red fluorescence). CX3CR1gfp fluorescent signals arose from resident microglial cells sparsely distributed along three retinal levels: at the ganglion cell, inner plexiform and outer plexiform layers (Fig. 4, green fluorescence).

Labeling with anti-mCherry antibody revealed weak immmunoreactivity in the inner nuclear layer and strong in the ganglion cell layer, consistent with the results obtained by *in situ* hybridization and analysis of the mCherry reporter natural fluorescence (Fig. S3).

## Discussion

In order to characterize changes in fractalkine (CX3CL1) expression in normal wt and with the progression of disease in rd10 mice, we compared retinas at seven different ages. At P5, when differentiation of retinal ganglion and amacrine cells and their ongoing programmed apoptosis and cell clearance takes place along with differentiation of horizontal cells, photoreceptors and formation of the outer plexiform layer, and initiation of conventional synapse production are almost complete in wt retina [52]–[54]. At P10, when the interconnections of vertical retinal networks are establishing, and bipolar cells are born and connections with ganglion cells are established [52], [54]; at eye-opening when synaptic ribbons begin to form and conventional synapses are produced at increased rate (P14) [52]–[54]; at the peak of rod photoreceptor death in rd10 [39], [51] and a sharp reduction in the rate of production of both ribbons and conventional synapses in wt (P22) [53]; at the initial disorganization of the loss in size of rod bipolar cell terminals in rd10 (P30) [45]; after near complete degeneration of rod photoreceptors, ongoing remodeling of AII amacrine cells, a key cell type in the primary rod circuit [42] resulting in near complete loss of rod bipolar cell responses in rd10 (P45) [44]; at the peak of cone photoreceptors degeneration and before cone bipolar cells and ganglion cells degenerate in rd10 retina (P60) [41], [43], [44].

Fractalkine protein was shown to exist as two functionally distinct forms: a cell-associated (membrane integrated) form and a soluble (cleaved) form that has been proposed to arise by processing of the membrane integrated protein [13], [22], . The ∼100 kDa CX3CL1 form found in our study, has been suggested previously to represent the mature full-length form and the only CX3CL1 form present at the cell surface, while the ∼85-kDa has been considered as a soluble CX3CL1 form. It was suggested that CX3CL1 is initially synthesized as a 50–75 kDa precursor, the form we did not detect in our immunoblots, that undergoes glycosylation and transport to the cell surface as a ∼100 kDa glycoprotein. This ∼100 kDa CX3CL1 form then could be released from the cell surface yielding a soluble ∼85 kDa fragment that likely contains the majority of the glycosylated ectodomain, and a ∼20 kDa transmembrane cytoplasmic domain fragment [22]. The membrane integrated ∼100-kDa band and a soluble ∼85-kDa CX3CL1 form were present in retinal lysates at P5, both in wt and rd10 retinas and at P45 and P60 in rd10 retinas only, indicating intensive CX3CL1 shedding. It has been suggested that the equilibrium between the membrane integrated and soluble forms of CX3CL1 plays an important role in regulating the biological activity of this molecule [57] and regulation of CX3CL1 cleavage may have a direct impact on neuronal survival both *in vitro* and *in vivo* [18], as evident with the injury of neurons or their exposure to glutamate, immediately leading to shedding of CX3CL1 [26], [57].

The ∼95 kDa protein band recognized by the same anti-chemokine domain antibody used in our study represents the most abundant band of CX3CL1 in both rd10 and wt retinas from P14 and the only protein form in wt retinas from P30 into young adulthood (P60). The ∼95 kDa protein accumulation may have resulted from recycling of the membrane integrated form, as fractalkine is a chemokine known to undergo internalization serving to protect the chemokine from degradation by metalloproteases [58], [59]. Indeed, mouse CX3CL1 was not only found at the cell surface and in juxtanuclear compartments, where it is presumably produced, but also in vesicle-like structures close to the cell membrane. These vesicles were positive for the early endosomal antigen 1 (EEA1) suggesting that CX3CL1 was internalized via the endosomal pathway [60]. These results are consistent with earlier reported findings that wild type CX3CL1 undergoes constitutive internalization via clathrin-coated pits and is recycled to the cell surface [58]. There is experimental evidence suggesting that the cytoplasmic domain of the protein is required for efficient internalization of CX3CL1 from the cell surface [59], [60]. Therefore we may expect that the internalized form of protein will have all the domains of the full-length protein and may migrate as ∼100 kDa form when extracted from the pool of endocytic vesicles in the retina lysates, or migrate slightly further, if it originates from a pool of the early endosomes, where it may undergo endogenous processing.

In accordance with the idea that neurons in the retina may accumulate pre-synthesized fractalkine, it has been previously described that in steady state, roughly half the total cellular pool of CX3CL1 is found intracellularly, and that the remaining fraction is on the plasma membrane as demonstrated for cultured cells of endothelial and epithelial origin, as well as for cultured fibroblasts [58], [59], [61], [62]. Indeed, in an earlier study, antibodies have been used that specifically recognize only the amino- or the carboxyl-terminal ends i.e., they allowed to distinguish between cleaved and full-length membrane integrated human fractalkine [63]. This property enabled to detect two closely migrating bands of similar size at ∼95 kDa within transfected cell lysates and a ∼85–90 kDa band that was found within the cell supernatants. According to the authors, the two closely migrating bands (∼95 kDa) represent different glycosylated cell-associated forms and the smaller band (∼85–90 kDa) indicates a cleaved soluble form of the molecule. Also, the two closely separated bands of ∼100 kDa corresponding to the cell-associated full-length mature fractalkine were clearly shown, but not described, in another study where the cellular processing of fractalkine and the mechanisms that lead to the release of the soluble ectodomain were investigated (see Fig. 1 C and Fig. 5 C in the publication [22]). On the other hand, in many other studies, a band of ∼95 kDa proved undetectable, possibly as a result of variations in the cell types and experimental conditions applied. The mechanism underlying the preferential localization of CX3CL1 under normal physiological conditions to the intracellular compartments in the mature retina is not clear.

Overall, in our study we show that there was a strong, positive correlation between relative band intensities of the membrane integrated and both, cleaved and presumably intraneuronal forms of fractalkine protein, suggesting the possibility that a dynamic equilibrium between the intracellular compartment and plasma membrane regulates the availability and processing of fractalkine at the cell surface.

Quantification of total CX3CL1 protein expression revealed comparable levels in retinas of rd10 and wt mice shortly after birth (P5). At later time-points, surprisingly from P10 that is before the onset of morphological changes in rd10 retinas, total CX3CL1 protein levels were significantly lower compared to wt retinas. Apparently, major neurochemical remodeling may occur prior to anatomical remodeling at an early stage of retinal degeneration, and likely accounting for the early decrease in fractalkine protein level. At later time-points, a further decrease in CX3CL1 total protein levels in rd10 retinas might be explained by a possible loss of neurons, eventually from the ganglion and inner nuclear cell layers, representing the main source of CX3CL1.

Although mRNA in degenerating rd10 retina is expressed at low levels, it seems to be stable and efficiently translated. Changes in the levels of CX3CL1 mRNA in rd10 and wt retinas detected by real-time PCR in our study, largely coincided with changes of CX3CL1 total protein expression, with the exception of P10, where no differences in mRNA levels between rd10 and wt mice have yet been detected. At P14 to P60, when change in mRNA levels correlate with change in protein level, we could propose that regulation of fractalkine gene expression is tightly controlled at the levels upstream of translation.

In rd1 mice, a model of RP bearing a point mutation of the β phosphodiesterase gene in exon 7 and representing a more severe form of RP, no significant changes in the levels of CX3CL1 mRNA during rod photoreceptor degeneration were detected [64]. The determination of CX3CL1 mRNA expression in this study was however accomplished using primers not separated by introns and by a semi-quantitative PCR method only.

However, analysis of mRNA expression level due to the posttranslational modifications cannot predict amount of the functional CX3CL1 protein it encodes for. Importantly, the identification of fractalkine forms in physiological and pathological *in vivo* settings forms the ultimate proof that posttranslational modification of the chemokine is relevant in regulating the biological activity of this molecule.

The fractalkine signaling and survival system seems to be regulated independently of the rod photoreceptor death; since changes in expression levels of fractalkine mRNA and total protein preceded the onset of degeneration (P16–18), while protein regulation toward extensive production of soluble fractalkine occurred at the late phase of rod degeneration (P30–45).

Our data collectively indicate that fractalkine is distinctively expressed and regulated in rd10 denegerative retinas compared to wild type retinas. Moreover, fractalkine protein is distinctively regulated in healthy mature neural retina (P30–P60), with ∼95-kDa being the principal form compared to healthy brain in particular, hippocampus, with ∼100, ∼95 and ∼85 kDa forms present. Notably, soluble fractalkine was found in early postnatal retina (P5) and adult (P200) dentate gyrus, suggesting that soluble form is required for the proper neurogenesis in both organs. Significantly, neurons in the retina release increased levels of soluble fractalkine when their health is compromised (P45–60).

Our data also suggest that the CX3CL1- CX3CR1 axis does not seemingly respond to any form of photoreceptor death (e.g. programmed cell death during development, rod and cone death during degeneration). It rather appears post-translationally regulated to modify the biological activity of CX3CL1 in case of pathological changes possibly affecting only the neurons producing fractalkine themselves. Indeed, programmed apoptosis and cell clearance of ganglion and amacrine cells during postnatal retinal development at around P5 and progressing remodeling of the inner retina between P30 into young adulthood (P60) do correlate with distinguished regulation of this signaling system suggesting that fractalkine may control a survival pathway to support cell and tissue integrity in times of need revealing a protective function of microglial cells.

Interestingly, the reduction in fractalkine levels occurred several days before the onset of rod photoreceptor death and weeks before morphological signs of bipolar, amacrine, ganglion cells degeneration, suggesting that fractalkine regulation is a dynamic process rather than a nonspecific consequence of neuronal death and of changes in the microenvironment.

In the present study we further demonstrated by *in situ* hybridization histochemistry, presence of CX3CL1 mRNA in subpopulations of neurons of the inner nuclear layer and layer of ganglion cells. Moreover, the distribution pattern of the Cherry reporter positive cells in the retina of CX3CL1cherry∶CX3CR1gfp mice was consistent with the outcome of our *in situ* hybridization studies. As pointed out in earlier study [20], the expression of the cherry and gfp transgenes in CX3CL1cherry∶CX3CR1gfp mice reports on the respective promoter activities but not the actual presence of the receptor/ligands, the fact particularly relevant with respect to axon and dendrite-bearing neurons. In this respect we may hypothesize that CX3CL1 protein may be expressed largely in the dendrites of the neurons in the inner nuclear layer, in particular, in the dendrites of bipolar cells (outermost area of the INL). Indeed, recent studies [7], [8] demonstrated a role of fractalkine in interaction of microglia with dendritic synapses in the CNS. Therefore, it is plausible to suggest that we may observe strong CX3CL1 mRNA labeling in many neurons but the cherry protein is expressed only in a few cell bodies in the INL of retina. It is likely, that these cells represent amacrine and bipolar cell subpopulations in both marginal-subtiers of the inner nuclear layer (INL), and a subpopulation of ganglion cells in the ganglion cell layer. However, the fact that displaced amacrine cells exist in the ganglion cell layer [65]–[67] as well as a displaced ganglion cell population in the inner nuclear layer [68] complicates interpretation. Further studies will be needed to determine the exact types of neurons expressing CX3CL1 in the mouse retina. Our data, however, do not demonstrate detectable CX3CL1 expression in the microvascular endothelial cells of the retinas of either rd10 or wt mice, contrary to the situation reported for retinal explants of human eyes [69].

The important question what role each form of the fractalkine protein may play under normal and pathological conditions also remains to be answered. Recent investigations in CX3CR1-deficient animals showed a negative effect of this mutation for adult neurogenesis and hippocampal circuit integrity [38], [70]. Mice lacking CX3CR1 have a significant reduction in the density of microglia during the postnatal period and exhibit transient defects in synaptic connectivity and plasticity in the postnatal hippocampus [7]. In accordance, another study further found that CX3CR1-deficient mice display significant cognitive impairment [71].

In the current literature the overall idea is that neurons control microglial activity and a lack of CX3CL1-CX3CR1 interaction leads to the “hyperactivity” of microglia unleashing potential neurotoxic properties. However, the protective/neurotoxic role of fractalkine and its receptor signaling in neurodegenerative disease is an intricate and highly debated research topic and it is becoming even more complicated as new studies reveal conflicting results depending on the CNS insult [72]–[75]. Very recent data show, however, protective effects of the fractalkine protein on retinal structure and function. This is supported by the analysis of light-injured rat retinas with transplanted mesenchymal stem cells engineered to secrete CX3CL1 [76]. Here the CX3CL1-expressing mesenchymal stem cells inhibited apoptosis of the retinal cells and microglial activation. Additionally, as shown during the revision of our manuscript, Peng et al. [77] demonstrated, that microglia activation was an early alteration in RP retinas in rd10 mice and that inhibition of microglia activation by minocycline through both anti-inflammatory and anti-apoptotic mechanisms reduced photoreceptor apoptosis, significantly protecting retinal structure, function and visual behavior. Furthermore, CX3CR1 deficiency in double knockout Rd10/CX3CR1-/- mice dysregulated microglia activation and subsequently resulted in increased photoreceptor vulnerability. Altogether these results suggest that the CX3CL1/CX3CR1 signaling pathway might protect against microglia neurotoxicity.

In summary, our results demonstrate that fractalkine ligand is differentially expressed under physiological and neurodegenerative conditions. It is continuously expressed by subpopulations of retinal neurons and exists at least in three different forms. The individual signaling functions of each CX3CL1 isoform in the retina should become a topic for future research.

## Supporting Information

Figure S1 **Control experiments of anti-CX3CL1 antibody signal specificity (A-F) and sex-related differences (G-L).** (A). Representative example of Western blots of rd10 retina at P60 (left lane) and wt mouse dentate gyrus at P200 (positive control, right lane) showing the three bands of CX3CL1 protein: 100-kDa, 95-kDa and 85-kDa. (B). 10 ng of recombinant human peptide (aa 25-100) migrated as a 70 kDa band. (C-E). Antigen-peptide neutralization (competition) assay. (C). Upon control peptide addition, a 95-kDa band is completely depleted in wt retina sample at P45. (D). 100-kDa, 95-kDa and 85-kDa bands are completely depleted in rd10 retina sample at P45. (E). Band 85-kDa is depleted and bands 100-kDa and 95-kDa are significantly diminished in wt mouse dentate gyrus sample at P200. – no peptide (left lanes), + peptide (right lanes). (F). No primary antibody control. (G-L). Representative examples of the Western blots of wt and rd10 retinas at P22 and P45. No sex-related differences in signal pattern of both specific and nonspecific bands were observed at all age groups examined. F-female, M-male.
(TIF)

Figure S2 **Western blot analyses of glyceraldehyde-3-phosphate dehydrogenase (GAPDH) protein expression in rd10 and wt mouse neuroretina.** Relative intensity of GAPDH signal detected by loading 10 µg of total protein per lane in wt and rd10 retina lysates at P5 to P60. Values (mean ± SEM, n = 5) are expressed in densitometric arbitrary units. Ns - difference is not statistically significant. Data are representative of five experiments.
(TIF)

Figure S3 **Representative cryostat section of CX3CL1cherry: CX3CR1gfp transgenic adult mouse retina immunostained with antibodies against mCherry (green) and GFP (red).** Dual immunoreactivity (A) showing the mCherry signal in inner nuclear (INL) and ganglion cell (GCL) layers, largely coinciding with results obtained by *in situ* hybridization studies, shown here as insert of rd10 section at P22 (B). CX3CLl/Cherry detected in FITC channel (C). Cryosections were counterstained with DAPI nucleic acid stain (D). CX3CL1/GFP detected in rhodamine channel (E). Outer nuclear (ONL) and inner plexiform (IPL) layers are devoid of specific signal. Of note, blood vessels are co-labeled due to mouse-on-mouse immunolabeling without blocking of endogenous IgGs. However, both primary and secondary antibodies controls exhibited no immunolabelling. Scale bars (A-E) represent 50 µm.
(TIF)
