# Supplementary figures and images for: CX3CL1 (Fractalkine) Protein Expression in Normal and Degenerating Mouse Retina: In Vivo Studies
Source: PLoS One. 2014 Sep 5;9(9):e106562. doi: 10.1371/journal.pone.0106562 (PMC4156323; doi:10.1371/journal.pone.0106562)

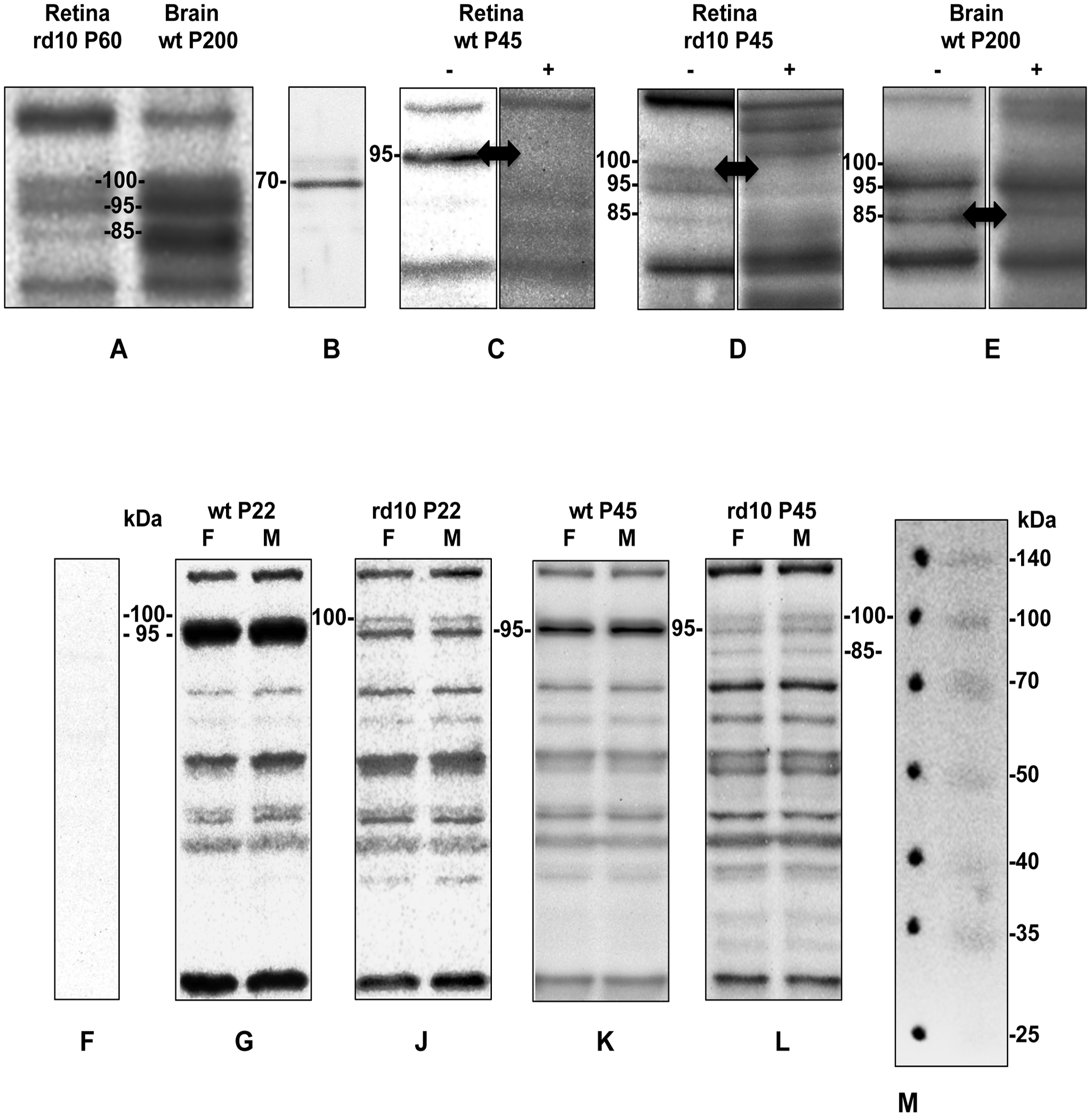

Supplement: Figure S1 — Control experiments of anti-CX3CL1 antibody signal specificity (A-F) and sex-related differences (G-L). (A). Representative example of Western blots of rd10 retina at P60 (left lane) and wt mouse dentate gyrus at P200 (positive control, right lane) showing the three bands of CX3CL1 protein: 100-kDa, 95-kDa and 85-kDa. (B). 10 ng of recombinant human peptide (aa 25-100) migrated as a 70 kDa band. (C-E). Antigen-peptide neutralization (competition) assay. (C). Upon control peptide addition, a 95-kDa band is completely depleted in wt retina sample at P45. (D). 100-kDa, 95-kDa and 85-kDa bands are completely depleted in rd10 retina sample at P45. (E). Band 85-kDa is depleted and bands 100-kDa and 95-kDa are significantly diminished in wt mouse dentate gyrus sample at P200. – no peptide (left lanes), + peptide (right lanes). (F). No primary antibody control. (G-L). Representative examples of the Western blots of wt and rd10 retinas at P22 and P45. No sex-related differences in signal pattern of both specific and nonspecific bands were observed at all age groups examined. F-female, M-male. (TIF) [file pone.0106562.s001.tif]

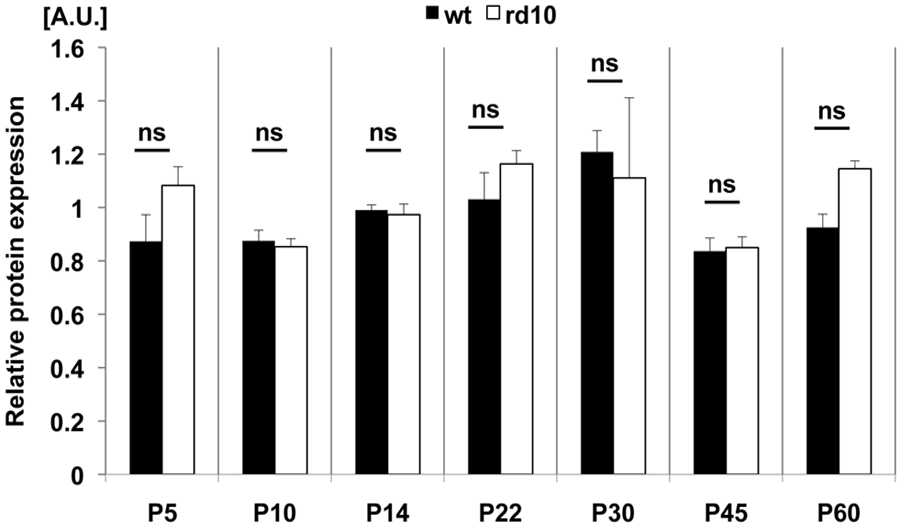

Supplement: Figure S2 — Western blot analyses of glyceraldehyde-3-phosphate dehydrogenase (GAPDH) protein expression in rd10 and wt mouse neuroretina. Relative intensity of GAPDH signal detected by loading 10 µg of total protein per lane in wt and rd10 retina lysates at P5 to P60. Values (mean ± SEM, n = 5) are expressed in densitometric arbitrary units. Ns - difference is not statistically significant. Data are representative of five experiments. (TIF) [file pone.0106562.s002.tif]

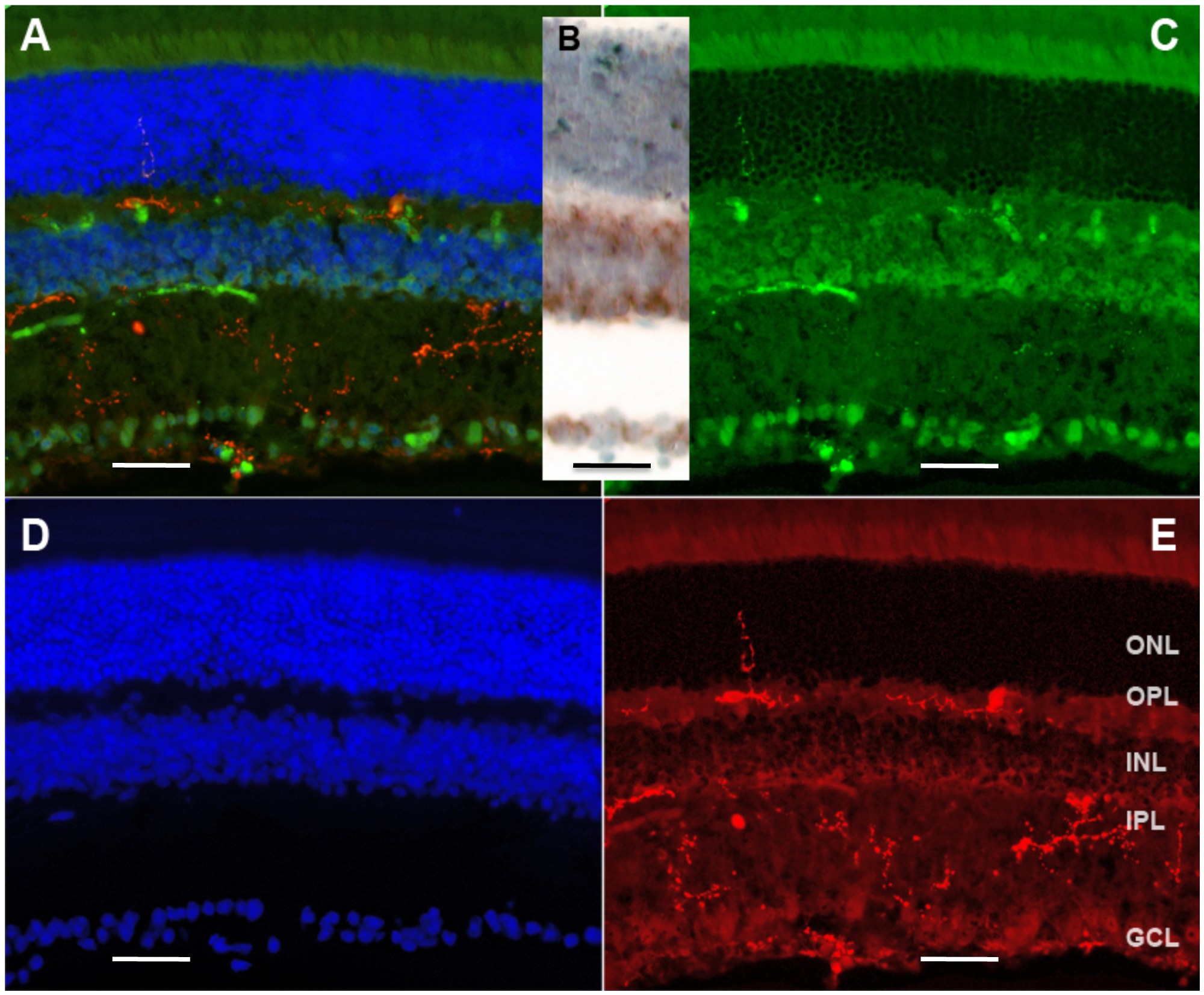

Supplement: Figure S3 — Representative cryostat section of CX3CL1cherry: CX3CR1gfp transgenic adult mouse retina immunostained with antibodies against mCherry (green) and GFP (red). Dual immunoreactivity (A) showing the mCherry signal in inner nuclear (INL) and ganglion cell (GCL) layers, largely coinciding with results obtained by in situ hybridization studies, shown here as insert of rd10 section at P22 (B). CX3CLl/Cherry detected in FITC channel (C). Cryosections were counterstained with DAPI nucleic acid stain (D). CX3CL1/GFP detected in rhodamine channel (E). Outer nuclear (ONL) and inner plexiform (IPL) layers are devoid of specific signal. Of note, blood vessels are co-labeled due to mouse-on-mouse immunolabeling without blocking of endogenous IgGs. However, both primary and secondary antibodies controls exhibited no immunolabelling. Scale bars (A-E) represent 50 µm. (TIF) [file pone.0106562.s003.tif]
